# Linked fire activity and climate whiplash in California during the early Holocene

**DOI:** 10.1038/s41467-022-34950-x

**Published:** 2022-11-23

**Authors:** Julia Homann, Jessica L. Oster, Cameron B. de Wet, Sebastian F. M. Breitenbach, Thorsten Hoffmann

**Affiliations:** 1 Department of Chemistry, Johannes Gutenberg-University Mainz, Mainz, Germany; 2 Department of Earth and Environmental Sciences, Vanderbilt University, Nashville, TN USA; 3 Department of Geography and Environmental Sciences, Northumbria University, Newcastle upon Tyne, UK

**Keywords:** Palaeoclimate, Biogeochemistry

## Abstract

Recent wildfire activity in semi-arid regions like western North America exceeds the range of historical records. High-resolution paleoclimate archives such as stalagmites could illuminate the link between hydroclimate, vegetation change, and fire activity in pre-anthropogenic climate states beyond the timescale of existing tree-ring records. Here we present an analysis of levoglucosan, a combustion-sensitive anhydrosugar, and lignin oxidation products (LOPs) in a stalagmite, reconstructing fire activity and vegetation composition in the California Coast Range across the 8.2 kyr event. Elevated levoglucosan concentrations suggest increased fire activity while altered LOP compositions indicate a shift toward more woody vegetation during the event. These changes are concurrent with increased hydroclimate volatility as shown by carbon and calcium isotope proxies. Together, these records suggest that climate whiplash (oscillations between extreme wetness and aridity) and fire activity in California, both projected to increase with anthropogenic climate change, were tightly coupled during the early Holocene.

## Abstract (web summary)

A California stalagmite records vegetation shifts and increased fire activity during the 8.2 kyr event. These changes occur alongside oscillations between wet and dry extremes suggesting tight coupling between climate whiplash and fire activity.

## Introduction

Increasingly, observations suggest that California’s hydroclimate is shifting toward shorter rainy seasons driven in particular by a decline in autumn precipitation^1,2^. The delayed wet season enhances moisture stress on living vegetation and further decreases moisture levels in dormant and dead vegetation, increasing flammability in a critical season when offshore winds intensify across the state^2,3^. These observations are corroborated by climate models which project further ‘sharpening’ of the winter rainy season with anthropogenic warming^2,4,5^, and increases in year-to-year precipitation volatility, or ‘climate whiplash’, with extreme wetness following extended droughts^6^. These trends can exacerbate fire recurrence by intensifying autumn dryness and promoting the growth of fast-responding grasses and brush during wet winter peaks that provide fuel to subsequent autumn wildfires^7–9^. Likewise, extreme rainfall events following fires are also projected to increase in high emissions scenarios, exacerbating threats of debris flows and vegetation loss^10^.

Speleothems host numerous isotopic and geochemical proxies that are capable of recording changes in precipitation amount, source, and seasonality that can shed light on hydroclimatic variability during key intervals of Earth’s past. Stalagmite-based multi-proxy records have documented a highly dynamic hydroclimate in California across the last several thousand years^11–15^. At White Moon Cave (WMC) in the California Coast Range, published stable carbon isotope (δ^13^C), calcium isotope (δ^44^Ca), and trace element (P/Ca) records in stalagmite WMC1 suggest an interval of enhanced precipitation volatility occurred between ~8250 and 8100 years BP (where present is 1950 CE), during the 8.2 kyr event, a pronounced Holocene cold period first noted in Greenland ice cores^13,15^. Furthermore, these proxies record a precursor event at 8300 years BP, potentially associated with an earlier pulse of freshwater from glacial Lake Agassiz^16^ when precipitation exceeded the wet and dry extremes of the last 60 years in coastal California^13,15^. Thus, hydroclimatically sensitive proxies from fast-growing stalagmites can provide evidence of changes in climate whiplash, but our understanding of how wildfires may be recorded in stalagmites is just beginning to emerge^17–19^. New proxies are necessary to document wildfire activity to investigate the relationship between climate whiplash and wildfire in the past.

We analysed levoglucosan and lignin as indicators of fire activity and vegetation composition in the portion of stalagmite WMC1 that brackets the 8.2 kyr event (from ~8600 to 6900 years BP) where previously analysed proxies suggest increased climate whiplash^13,15^. The anhydrosugar levoglucosan naturally originates only from the combustion of cellulose^20^, where it is emitted into the particle phase and constitutes a fingerprint of biomass burning^21,22^. While already used in other paleoclimate archives, especially in aerosols^23–26^, neither levoglucosan nor its isomers are typically analysed in speleothems. Lignin, a biopolymer found exclusively in vascular plants, consists of sinapyl-, coniferyl-, and coumaryl alcohol units linked by stable ether and C-C bonds^27^. For analysis, lignin is broken into monomeric units by oxidative digestion. The resulting lignin oxidation products (LOPs) can be divided into three groups according to their phenolic structures: the vanillyl group (V), the syringyl group (S), and the cinnamyl group (C) (Supplementary Fig. 1)^28^. Through comparison of the C/V and S/V ratios, we can determine if the lignin originated from angiosperms or gymnosperms and distinguish woody and non-woody vegetation^29^.

WMC, located in the Santa Cruz Mountains east of Davenport, CA (N37°00′, W122°11′) experiences a warm-summer Mediterranean climate. Local climate and geology of the site are fully described in ref. 15. Vegetation directly above WMC consists of mixed evergreen forest composed of coastal Redwood and Douglas Fir with interspersed Coyote Bush and grasses. The site burned in August 2020 during the CZU Lightning Complex Fire which covered 350 km^2^ and began as the Warnella Fire near Davenport, CA^30^. Smaller fires frequently occur in the surrounding coast range during most fire seasons.

Here we demonstrate a link between fire activity, vegetation change, and climate whiplash in California during the early Holocene. We also provide an analysis of levoglucosan and LOPs in modern dripwater and calcite to place constraints on how surface signals of fire and vegetation move through the modern cave system.

## Results

### Stalagmite-based fire and vegetation proxies

**Fig. 1:**
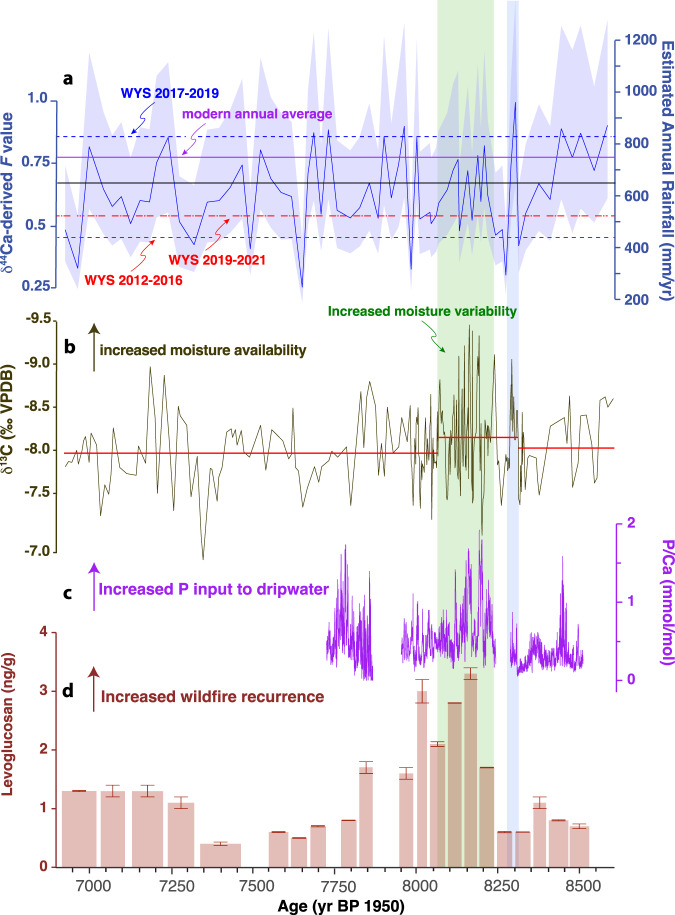
Proxy records for White Moon Cave (WMC) stalagmite. **a** Fraction of calcite remaining in solution (F) computed from speleothem δ^44^Ca using Rayleigh fractionation model^15^ and translated to estimated rainfall using modern cave system δ^44^Ca values and modern rainfall. Horizontal lines provide a comparison to rainfall amounts in the last decade: Wys (water years; 12-month period from 1 October to 30 September designated by the calendar year in which it ends) 2012–2016 (red dashed, 58% modern); Wys 2017–2019 (blue dash-dot, 109% modern); Wys 2019–2021 (red dash-dot, 69% of modern) modern average annual (1950–2021, purple); WMC1 record average computed rainfall (black). **b** δ^13^C (‰ Vienna Peedee Belemnite (VPDB)), red lines highlight mean values of segments between changepoints identified based on mean and variance. **c** P/Ca (mmol mol^−1^). **d** Levoglucosan concentrations (ng g^−1^). Error bars represent the standard deviation of samples measured in duplicate. Vertical shading delineates the duration of the 8.2 kyr event (green) and precursor event (blue).

**Fig. 2:**
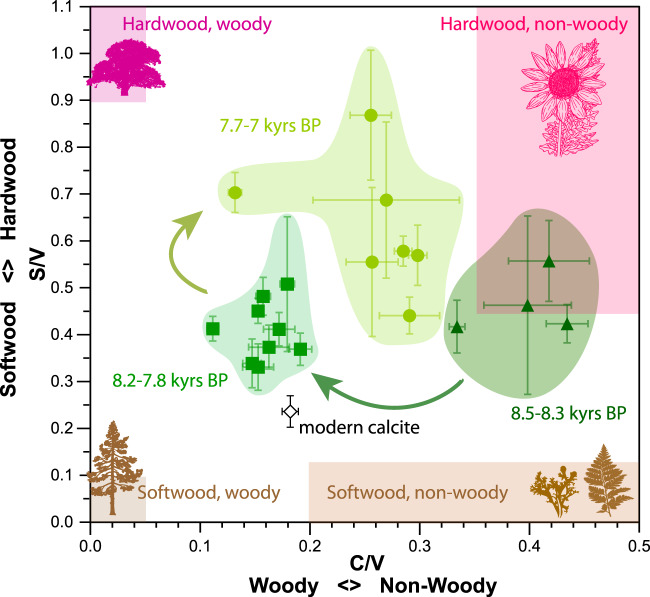
Lignin oxidation product (LOP) ratios (S/V versus C/V) for speleothem samples and modern calcite. Shown are speleothem samples with ages from 8.5 to 8.3 kiloyears before present (kyrs BP) (triangles), 8.2 to 7.8 kyrs BP (squares), and 7.7 to 7.0 kyrs BP (circles). The combined modern calcite samples are shown as an open diamond. The area shaded in dark brown corresponds to lignin originating from gymnosperm woody plants, as defined by Hedges and Mann^29^. The orange area is associated with gymnosperm non-woody plants, the purple area corresponds to angiosperm woody samples, and the pink area is associated with angiosperm non-woody plant parts. Error bars represent the standard deviation of samples measured in duplicate. The arrows highlight the changes over time. Plant icons available from phyloPic.org^60^ (lower left) and freesvg.org^61^ (others) and are licensed under Public Domain Dedication 1.0^62^.

Speleothem calcite deposited between 8217 ± 23 and 7847 ± 20 years BP shows elevated levoglucosan levels relative to the remaining stalagmite record (Fig. 1d and Supplementary Table 1), with the highest concentrations found between 8167 ± 20 and 8019 ± 15 years BP. This suggests that fire activity in the vicinity of WMC was elevated during the 8.2 kyr event relative to the rest of this early-to-mid-Holocene record (8500–6900 years BP). Unlike levoglucosan concentrations, we find no clear pattern in the concentration of LOPs across the speleothem record (Supplementary Figs. 2 and 4 and Supplementary Table 1). However, when plotted by their C/V and S/V ratios (Fig. 2) the speleothem samples fall into three distinct zones suggesting that the vegetation community surrounding the cave changed over time. Most prominently, between 8300 and 8200 years BP, we observe a shift from non-woody to woody vegetation. This is followed by a shift back to more non-woody vegetation between 7800 and 7700 years BP. The period of more woody vegetation (specifically 8271 ± 23 to 7793 ± 25 years BP) includes the interval of elevated levoglucosan concentrations (8217 ± 23 to 7847 ± 20 years BP).

The recovery of the ^13^C_6_-levoglucosan spike for all but one stalagmite sample fall within the standard deviation of the average recovery of the whole sample set (Supplementary Fig. 2). This includes the samples with the highest measured levoglucosan concentrations, indicating that there is no divergent recovery rate and that these high values are unlikely to be an analytical artefact. Theoretically, stalagmite levoglucosan concentrations could also reflect changes in subsurface seepage water flow pathways that would route water from different locations on the surface to the stalagmite. Changes in subsurface water routing would also likely bring water into contact with different mineral phases within the host rock, and thus should be recorded by stalagmite strontium isotope ratios (^87^Sr/^86^Sr) which reflect the balance of water interactions with soils and host rocks with different ^87^Sr/^86^Sr fingerprints. Although we do observe host rock and soil phases with different ^87^Sr/^86^Sr values above WMC, we do not find a shift in stalagmite ^87^Sr/^86^Sr during the 8.2 kyr event^15^ concurrent with the levoglucosan peak. This indicates that a sustained change in seepage water routing is very unlikely during this interval. Thus, the observed changes in levoglucosan concentrations and LOP ratios across the WMC1 record most likely reflect shifts in fire activity and vegetation community in the vicinity of the cave across the 8.2 kyr event.

Increased fire activity coincident with higher proportions of woody vegetation is also evident in Holocene charcoal and pollen records from across the Pacific Northwest^31^, reflecting a potential feedback of increased woody fuel driving fire dynamics or a climate driver that affects both vegetation type and fire activity. The vegetation change noted in WMC1 is broadly consistent with regional changes in vegetation as recorded by pollen from the marine sediment core ODP 1018 off the coast of Santa Cruz which indicates increased redwood and decreased herbs and chaparral species just prior to 8000 years BP^32^. Lake sediment records from California and southern Oregon suggest increasing fire activity under progressively more intense aridity from the early to middle Holocene^33–36^, with lakes in the Klamath Mountains suggesting enhanced fire activity at 8400 years BP and the growth of a chaparral understory through the mid-Holocene^37^. However, challenging chronologies and low sediment accumulation rates preclude investigation of fire-vegetation relationships at these sites at temporal resolution comparable to the WMC speleothem.

### Fire and vegetation proxies in the modern cave environment

To explore the behaviour of levoglucosan and LOPs in the modern cave environment in support of our interpretations of the paleoenvironmental record, we analysed glass plate calcites and dripwaters from several locations within the cave. Glass plates containing ~3 years of calcite growth (see Methods section) were analysed from sites WMC1 and WMC2 in the upper level of the cave (Supplementary Fig. 3). Levoglucosan concentrations in these modern calcites (3.6 ± 0.3 ng ∙ g^−1^ and 6.6 ± 0.3 ng ∙ g^−1^) are higher than all early to mid-Holocene speleothem samples. In addition, we analysed portions of the speleothem younger than 6900 years BP, and these showed higher levoglucosan concentrations up to 4.7 ± 0.3 ng ∙ g^−1^ (Supplementary Table 1). This increase towards the present may be indicative of higher levoglucosan input into the soil through increased fire activity in the mid to late Holocene, consistent with regional lake records^33,35,36^. However, we do not have precise age constraints on this part of the stalagmite and can only estimate their ages to be between 6900 and ~240 years BP. Like levoglucosan, modern calcite samples show higher LOP concentrations than speleothem samples (Supplementary Figs. 2 and 4). This might reflect a denser present-day vegetation above the cave compared to the early Holocene. The C/V and S/V ratios for modern calcite samples are consistent with the current mixed evergreen forest above the cave and are most similar to the speleothem values from ~8200 to 7800 years BP when the plant community above the cave consisted of more woody vegetation (Fig. 2).

**Fig. 3:**
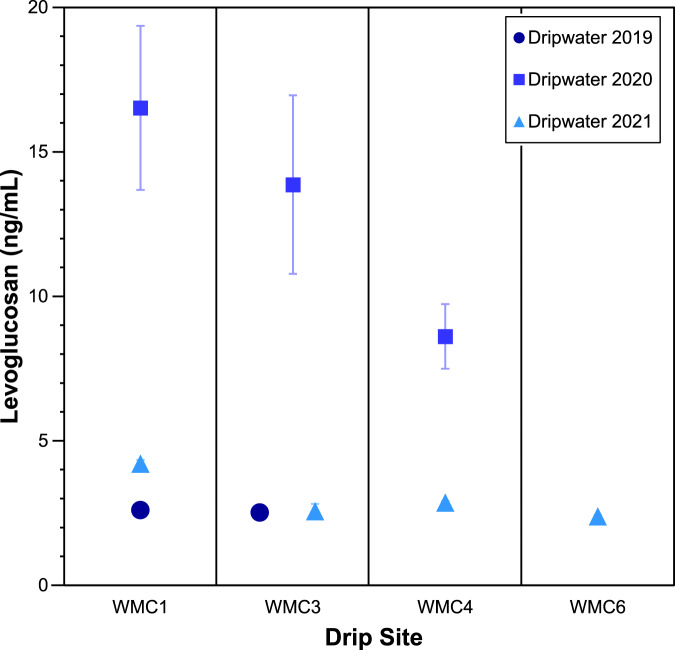
Levoglucosan concentrations in dripwater. Water samples were collected in February 2019, February 2020, and December 2021. Error bars represent the standard deviation of samples measured in duplicate and may be smaller than the symbols.

To investigate in-cave spatial and temporal patterns in levoglucosan concentrations and their relationships to recent fire activity, we analysed dripwater from four sites collected during the wet seasons (December to February) of 2019, 2020, and 2021 (Fig. 3). Waters from sites WMC1 and 3 in the upper level of the cave had higher levoglucosan concentrations than waters collected from sites WMC4 and 6, which are approximately 55 m deeper below the surface (Supplementary Fig. 3). These differences may reflect increased adsorption of levoglucosan to rock surfaces along longer seepage water flow paths. Notably, levoglucosan levels of all samples obtained in February 2020 are significantly higher than in samples from February 2019 and December 2021. Smaller (<1 km^2^) fires occurred within 30 km of the cave in autumn 2019^38^. Levoglucosan can easily be transported in the particle phase over such distances^24,39^ although it is currently unclear how signal intensity changes in relation to the distance to the fire. Winter rainfall prior to dripwater collection (January-February) was much higher in 2019 than in 2020 (430 vs. 75 mm)^40^. This might have supported leaching of levoglucosan from the soil in 2019 and resulting loss of signal from previous fire seasons. The samples collected in December 2021, after the August 2020 fire, do not show elevated levoglucosan levels relative to other years. Like 2019, the interval prior to water sampling in December 2021 was particularly wet (280 mm, October–December 2021) and more than one rainy season had passed between fire occurrence and dripwater collection. This suggests that the levoglucosan signal in WMC dripwater may only reflect the fire activity of the most recent fire season, and that high rainfall intervals rapidly leach the signal from the soil. Further investigation into the persistence of the levoglucosan signal in cave environments and the required proximity of fires for influencing dripwater chemistry is warranted to better understand the dynamics of this proxy. Given a growth rate of ~0.1 mm yr^−1^ of stalagmite WMC1 and the sampling resolution of the levoglucosan analyses (5–9 mm or ca. 50–90 years), we are not able to evaluate interannual variability in fire activity in the early Holocene. However, peak levoglucosan levels over 4 samples in stalagmite WMC1 indicates that periods of elevated fire activity persisted above the cave over ~150 years during the 8.2 kyr event and that fire activity in this interval exceeded that in the remaining record from 8600 to 6900 years BP.

## Discussion

Tree-ring data from western North America reveal strong sensitivity of fire activity to summer temperatures and hydrological drought since the late Holocene^41–43^. In the southern Cascades^43^ and Sierra Nevada^44^, variations in fire activity have been associated with changes in the magnitude of interannual fluctuations in rainfall amount, with enhanced fire activity occurring when high interannual variations in the Palmer Drought Severity Index (PDSI) coincide with higher temperatures^43^. Similar relationships are evident in the early to mid-Holocene record of the Pacific Northwest, where pollen and charcoal reconstructions link increased fire activity to summer drought^31,33^, while lacustrine oxygen isotope records document wetter winters^45^.

**Fig. 4:**
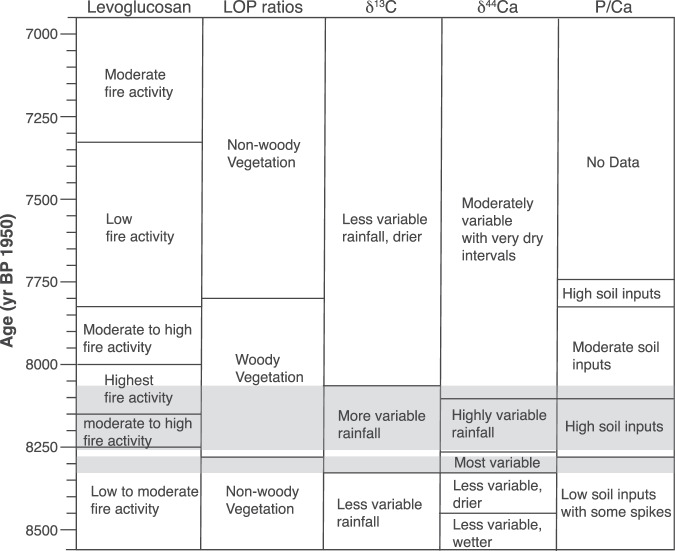
Summary of proxy interpretations for stalagmite WMC1 with time. Grey shading denotes the precursor event (centered on 8300 years BP) and the 8.2 kyr event. See Fig. 1 for proxy timeseries.

Winter rainfall accounts for >80% of annual precipitation at WMC^15^. Thus, stalagmite precipitation proxies will provide a record of winter rainfall changes, while the levoglucosan record likely reflects fire activity from the preceding summer. During the 8.2 kyr event and a ‘precursor’ event at 8300 years BP, stalagmite isotope and trace element proxies all indicate overall wetter winters and a potentially more volatile hydroclimate relative to the rest of the record^13,15^ (Figs. 1 and 4). Semi-quantitative estimates of precipitation determined using δ^44^Ca, a proxy that is uniquely sensitive to prior carbonate precipitation (PCP), suggest that during the 8.2 kyr event rainfall oscillated between dry and wet periods with similar magnitude as over the instrumental record (1950–2020) (Fig. 1a). However, during the precursor event, these oscillations exceeded modern multi-year droughts and precipitation excesses, including the recent 2012–2015 multi-year drought^46^ and ensuing 2016–2017 wet interval^15^. Similarly, the higher resolution δ^13^C record is sensitive to changes in PCP via preferential removal of ^12^C during CO_2_ degassing from seepage waters and changes in soil respiration driven by precipitation or temperature changes^13,47^. Changepoint analysis conducted on the δ^13^C record (Fig. 1b) suggests a shift in mean and variance at the onset of the precursor event, and another shift at the end of the 8.2 kyr event interval at ~8070 years BP. This indicates the combined precursor and 8.2 kyr event were characterised by higher variance and lower mean δ^13^C compared to the Holocene. This agrees with overall wetter (lower δ^13^C) but more volatile winter conditions. The changepoint identified in the δ^13^C record just predates the rise in levoglucosan near the onset of the 8.2 kyr event at 8217 ± 23 years BP (Figs. 1 and 4). The vegetation shift indicated by LOP ratios also occurs at 8271 ± 23 years BP, just after the precursor event but preceding the rise in levoglucosan. This indicates that abrupt changes from wet to dry extremes during the precursor event may have initiated a shift towards more woody vegetation that persisted as fire activity increased and may have provided increased fire fuel^31^. Notably, stalagmite P/Ca (Fig. 1c), which reflects inputs of soil colloidal material to dripwater^15^ and has been linked with enhanced fire activity in Australian stalagmites^19^, also increases at this time.

These proxy comparisons describe a link between hydroclimate volatility, vegetation change, and fire occurrence during the 8.2 kyr event similar to that observed in modern tree-ring records that are of higher temporal resolution than the stalagmite, and in Holocene lake sediments that are of lower resolution^31,33,43,44^. Seasonality was enhanced in the early Holocene including during the 8.2 kyr event, with increased summer insolation leading to higher summer temperatures and vice versa^36,45^. Although freshwater forcing led to cooling in the North Atlantic region, modelling indicates only a small (<0.5 °C) reduction in average annual temperatures associated with the 8.2 kyr event in western North America^48^. Similar to other instances of freshwater release to the North Atlantic^11,49,50^ the effect of the 8.2 kyr event on California climate was primarily through hydroclimatic change. The WMC1 δ^13^C record resolves precipitation changes internal to the 8.2 kyr event and suggests that the interval was characterised by heightened interannual precipitation volatility even though the mean shift in precipitation was small. Similar to links between modern PDSI variability and summer temperatures^43^, we propose that this heightened volatility coupled with the enhanced early Holocene summer temperatures^36^ drove the shift to more woody vegetation and elevated fire activity.

Our method for the quantification of levoglucosan and LOPs in small speleothem samples (<1 g) and dripwater offers a pathway for reconstructing wildfire activity and paleo-vegetation, respectively in paleoclimate archives on longer pre-instrumental timescales than what is offered by tree-ring records and at higher temporal resolution than what is offered by lake sediments. Further information from cave monitoring studies along geologic, ecologic, and hydrologic gradients is necessary to evaluate the degree to which levoglucosan records more distal fires, the longevity of the levoglucosan signals in soils, and how the signals are transported through the epikarst and embedded in speleothems. Study sites with a known fire history or with adjacent paleoclimate archives containing other fire and vegetation markers like fire scars in tree-rings, or charcoal, pollen, or polycyclic aromatic hydrocarbons (PAHs) in lake sediments should be prioritised to better restrict the parameters that influence the levoglucosan and LOP signal in speleothems. Techniques to analyse PAHs in dripwaters^51^ and speleothems^52,53^ are also in development. Combined analysis of levoglucosan and PAHs within the same speleothem would offer another powerful tool for disentangling the relationships between climate, fire, and vegetation change on multiple timescales.

The combined records of hydroclimatic change, wildfire, and vegetation community from WMC support the link between precipitation volatility and fire activity in western North America that has been observed in tree-ring records in the late Holocene, inferred from mid-Holocene lake records, and predicted for future warming scenarios^7,8^. Our record provides evidence for this climate-fire relationship under early Holocene boundary conditions prior to substantial forcing from anthropogenic greenhouse gas emissions and land use changes such as fire suppression^54^. Furthermore, our records suggest enhanced volatility and fire activity during the 8.2 kyr event, an interval of freshwater forcing and abrupt cooling in the North Atlantic. The incorporation of paleo-fire and vegetation proxies into the WMC1 record demonstrates the potential for fast-growing speleothems from fire-prone regions to illuminate the drivers behind precipitation volatility and the relationship between ‘climate whiplash’ and fire under a range of background climate states.

## Methods

### Statistical analysis of previously sampled proxy records

In order to statistically identify changes in the amplitude and frequency of proxy variability, and thus to pinpoint the potential onset and cessation of changes in hydroclimate volatility, we conducted changepoint analysis on the previously published stalagmite δ^13^C record^13^ using the changepoint package in R^55^. Changepoints were identified in the δ^13^C record as changes in mean and variance using the PELT algorithm^56^ and the modified Bayes Information Criterion (MBIC) penalty^57^. The δ^13^C timeseries was chosen for this analysis because of its continuity (trace element records for this stalagmite have gaps) and high resolution (the δ^44^Ca record is of much lower temporal resolution).

An age model was constructed for the levoglucosan and LOP samples using the Stalage algorithm in R^58^ and the previously collected stalagmite ^230^Th/U dates^13^. The ages of the upper and lower surfaces of the segments collected for levoglucosan and LOP analysis were modelled separately and presented as the upper and lower bounds of the sample ages.

### Chemicals and materials

Analytical standards of levoglucosan (99%), acetosyringone (97%), acetovanillone (≥98%), para-coumaric acid (≥98%), ethylvanillin (99%), ferulic acid (99%), syringaldehyde (98%), and syringic acid (>95%), as well as L-ascorbic acid (SigmaUltra) were purchased from Sigma-Aldrich. An analytical standard of ^13^C_6_ levoglucosan (98%) was obtained from Cambridge Isotope Laboratories. An analytical standard of vanillin (99%) was obtained from Acros Organics, and an analytical standard of vanillic acid (98%) was obtained from Alfa Aesar. An analytical standard of ammonium acetate (≥99%) and ultrapure acetonitrile (ACN, LC/MS grade) were obtained from VWR Chemicals. Sodium hydroxide (pellets, ≥99%), hydrochloric acid (HCl, suprapure, 30%), and water (LC-MS grade) were purchased from Merck KGaA. Ammonium hydroxide solution (NH_3_, analytical grade, 25%) was obtained from Honeywell Fluka. Ultrapure methanol (MeOH, LC/MS grade) was obtained Carl Roth while ultrapure dichloromethane (DCM, LC-MS grade, ≥99.8%) was purchased from Fisher Scientific. Ultrapure water with 18.2 MΩ resistance was produced using a Milli-Q water system from Merck Millipore (Darmstadt, Germany). Solid-phase extraction columns (Oasis HLB, 6 mL tubes, 200 mg packing material and Oasis HLB, 1 mL tubes, 30 mg packing material) were purchased from Waters.

### Speleothem and modern calcite sample preparation

Stalagmite samples were obtained by using a tile saw to sequentially slice 0.5–0.9 cm segments along a section of the WMC1 growth axis. This resulted in calcite samples that ranged in weight from 0.5 to 1.3 g. The 20 speleothem samples were first cleaned in DCM/MeOH 9:1 by 10 min of sonification and subsequently washed with Milli-Q water. Afterwards, the samples were bedecked with HCl (0.6%) for 5 min to remove any adhesive contaminations, washed again with Milli-Q water, and dried at 150 °C. Thereafter, the sample blocks were pulverised using an electric coffee grinder (Grundig CM 4760 GMS2070).

Samples of modern calcite were scraped from the surface of artificial substrates (glass plates) that were placed in the cave underneath active drip sites. Artificial substrates were placed under drip site WMC2 in December 2015 and WMC1 in March 2016 in the upper level of the cave (see Supplementary Fig. 3). Both plates were recollected in June 2018.

For the extraction of levoglucosan, the pulverised speleothem and modern calcite samples were spiked with 100 µL of a solution of ^13^C_6_ levoglucosan (100 ng ∙ g^−1^ in ACN) and extracted twice with 5 or 2.5 mL of MeOH, respectively. The extraction was assisted by ultrasonic sound and lasted 45 min at a time. After sonification the samples were allowed to rest for 10–20 min to improve phase separation. Then, the supernatant was removed, filtered over a 1.0 µm glass fibre fabric filter (Macherey Nagel), and evaporated under a gentle stream of nitrogen at 30 °C. The residue was taken up in 200 µL of ACN/H_2_O (95:5) by 10 min of sonification at 45 °C. The solution was filtered with a 0.2 µm PA-filter (Altman-Analytik) and stored in the freezer at ‒25 °C.

For the analysis of LOPs, the extracted speleothem powder was dried overnight at 50 °C and dissolved in 3 mL HCl (30%) per g sample and then diluted 1:1 with Milli-Q water. The two modern calcite samples were combined to assure that the resulting LOP concentrations would be above LOD. The 200 mg HLB cartridges were preconditioned with 6 mL of each MeOH and ultrapure water, which was acidified to pH 1–2 with HCl (30%). The speleothem solution was filtered through paper filters (Whatman, Grade 40, 8 µm pore size) to prevent undissolved materials from clogging the cartridges and was loaded onto the cartridges using sample reservoirs. The Cartridges were washed twice with 6 mL of acidified ultrapure water and then dried by sucking air through the cartridges using a vacuum pump. The polymeric lignin was eluted using 10 portions of 500 µL MeOH. The solvent was subsequently evaporated under a gentle stream of nitrogen at 30 °C. The CuSO_4_ oxidation procedure was developed based on the method described by Yan and Kaiser (2018)^59^. The residue was dissolved in 200 µL MeOH and sonicated for 10 min at 45 °C. The solution was added to a PTFE microwave digestion vessel with a capacity of 500 µL. The procedure was repeated with 100 µL MeOH and the combined solutions evaporated to dryness under a gentle stream of nitrogen at 30 °C. The residuum was dissolved in 200 µL NaOH (1 mol ∙ mL^−1^) through sonification for 10 min at 45 °C. To this, 10 µL each of CuSO_4_ solution (10 mmol ∙L^−1^ in H_2_O) and a solution of L-ascorbic acid (0.2 mol ∙ L^−1^ in H_2_O) were added. The vessels were purged with nitrogen for 20 s each and quickly capped to ensure inert gas atmosphere. The digestion vessels were packed into a bigger Teflon vessel 5 at a time and covered with 7 mL of NaOH (1 mol ∙ mL^−1^) to ensure better heat transfer. These vessels were also purged with nitrogen for 1 min. The vessels were heated to 155 °C in 5 min and held at that temperature for 90 min using a 5890A Gas Chromatograph (Hewlett Peckard, USA). Immediately after opening the vessels, 10 µL of a 1 µg ∙ mL^−1^ standard solution of ethylvanillin in ACN was added as an internal standard and the oxidised sample solution was acidified to pH 1–2 with HCl (30%). The 30 mg HLB cartridges were preconditioned with two times 1 mL of each MeOH and ultrapure water, which was acidified to pH 1–2 with HCl (30%). The acidified oxidised sample solutions were loaded onto the cartridges and the vessels were washed with acidified water twice. Subsequently, the cartridges were washed twice with their void volume and then then dried by sucking air through the cartridges using a vacuum pump. The LOPs were eluted using 8 portions of 125 µL of ACN with 2% NH_3_ added to reach a basic pH of 8–9. The eluate was evaporated under a gentle stream of nitrogen at 30 °C and the residue was dissolved in 200 µL ACN/H_2_O (1:9) by 10 min of sonification at 45 °C. The solution was filtered with a 0.2 µm PA-filter (Altman-Analytik) and stored in the freezer at ‒25 °C.

Cave dripwater samples were collected from four active drip sites at different depths within the cave in February 2019 and 2020 and December 2021 (Supplementary Fig. 3). The samples were passed over a preconditioned 200 mg HLB cartridge (see above). The flow-through was collected and subsequently evaporated using a vacuum centrifuge (Eppendorf Concentrator plus, 248 g) at 30 °C. The centrifuge tubes were washed with two portions of 1.5 mL ACN each by 10 min of sonification at 30 °C. The combined solutions were evaporated under a gentle stream of nitrogen and the residue was taken up in 200 µL of ACN/H_2_O (95:5) by 10 min of sonification at 45 °C. The solution was filtered with a 0.2 µm PA-filter (Altman-Analytik) and stored in the freezer at ‒25 °C.

The loaded SPE cartridges were handled following the same procedure described for the speleothem samples in Preparation for lignin analysis.

### Levoglucosan analysis

All samples were analysed for levoglucosan and LOPs. For this, they were each measured in duplicate. The analysis was carried out on a Dionex UltiMate 3000 ultrahigh-performance liquid chromatography system (UHPLC) that was coupled to a heated electrospray ionisation source (HESI) and a Q Exactive Orbitrap high-resolution mass spectrometer (HRMS) (all by Thermo Fisher Scientific). To retard the analytes, an iHILIC-Fusion (+) column, 100 mm × 2.1 mm with 1.8 μm particle size (Hilicon), was used. The injection volume was 10 µL. A H_2_O/ACN gradient programme was applied at a flow of 0.4 mL ∙ min^−1^. The gradient started with 97% eluent B (100% ACN) and 3% eluent A (consisting of 5 mmol ∙ L^−1^ ammonium acetate in H_2_O). Eluent B was held at 97% for 0.5 min and then decreased to 90% within 0.5 min. Afterwards, eluent B was further decreased to 85% in 5 min and 60% in 1.5 min, respectively. Finally, it was decreased to 50% within 1.5 min, held for 2.0 min, and then eluent B was increased to the initial value of 97%. This was held for 8.5 min to re-equilibrate the column. To improve ionisation, a post-column flow of 50 mmol ∙ L^−1^ NH_4_OH in MeOH was applied with a flow rate of 0.1 mL ∙ min^−1^. The HESI source was operated in a negative mode so that deprotonated molecular ions [M-H]^-^ were formed. The spray voltage was −4.0 kV, the HESI probe was heated to 150 °C, and the capillary temperature was 350 °C. The sheath gas pressure was 60 psi and the auxiliary gas pressure was 20 psi. The mass spectrometer was operated in full scan mode with a resolution of 70,000 and a scan range of m = z 80–500. At the respective retention time windows, the full scan mode was alternated with a targeted MS^2^ mode with a resolution of 17,500. For the MS^2^ mode (i.e., parallel reaction monitoring mode in the software Xcalibur, provided by Thermo Fisher Scientific), higher-energy collisional dissociation (HCD) was used with 35% normalised collision energy (NCE).

### LOP analysis

The analysis of the LOPs was carried out on a Dionex UltiMate 3000 UHPLC that was coupled to a heated ESI and a Q Exactive Orbitrap HRMS (all by Thermo Fisher Scientific). To separate the LOPs, an Acquity UPLC CSH Fluoro Phenyl (PFP) column, 100 mm × 2.1 mm with 1.7 μm particle size (Waters), was used. The injection volume was 5 µL. A H_2_O/ACN gradient programme was applied with a flow of 0.5 mL ∙ min^−1^. The gradient started with 5% eluent B (consisting of 98% ACN and 2% H_2_O) and 95% eluent A (consisting of 98% H_2_O, 2% ACN, and 400 μL ∙ L^−1^ formic acid). Eluent B was increased to 10% within 0.5 min and held for 4.5 min. It was then increased to 15% within 1 min and further increased to 30% in 1 min and to 50% in 0.5 min, respectively. Finally, it was increased to 99% within 0.5 min, held for 1.5 min, and then eluent B was decreased to the initial value of 5%. The HESI source was operated in a negative mode so that deprotonated molecular ions [M-H]^−^ were formed. The spray voltage was −4.0 kV, the HESI probe was heated to 150 °C, and the capillary temperature was 350 °C. The sheath gas pressure was 60 psi and the auxiliary gas pressure was 20 psi. The mass spectrometer was operated in full scan mode with a resolution of 70,000 and a scan range of m = z 80–500. At the respective retention time windows, the full scan mode was alternated with a targeted MS^2^ mode with a resolution of 17,500. For the MS^2^ mode (i.e., parallel reaction monitoring mode in the software Xcalibur, provided by Thermo Fisher Scientific), HCD was used with 35% NCE for all analytes.

## Supplementary information

Supplementary Information

## Data Availability

The levoglucosan and LOP data generated for this study are provided in Supplementary Table 1 and are publicly archived with the National Centers for Environmental Information at https://www.ncei.noaa.gov/access/paleo-search/study/37018. Previously published proxy data are archived at https://www.ncei.noaa.gov/access/paleo-search/study/32012 and https://www.ncei.noaa.gov/access/paleo-search/study/22270.
