# Supplementary material for: Linked fire activity and climate whiplash in California during the early Holocene
Source: Nat Commun. 2022 Nov 23;13:7175. doi: 10.1038/s41467-022-34950-x (PMC9684419; doi:10.1038/s41467-022-34950-x)
Supplement: Supplementary file 1 — Supplementary Information [file 41467_2022_34950_MOESM1_ESM.pdf]

Supplementary information

Linked fire activity and climate whiplash in California during the early Holocene

Julia Homann[1], Jessica L. Oster*[2], Cameron B. de Wet[2], Sebastian F.M. Breitenbach[3], Thorsten Hoffmann[1]

[1] Department of Chemistry, Johannes Gutenberg-University Mainz, Germany

[2] Department of Earth and Environmental Sciences, Vanderbilt University, Nashville, USA

[3] Department of Geography and Environmental Sciences, Northumbria University, Newcastle upon Tyne, UK

*corresponding author: jessica.l.oster@vanderbilt.edu

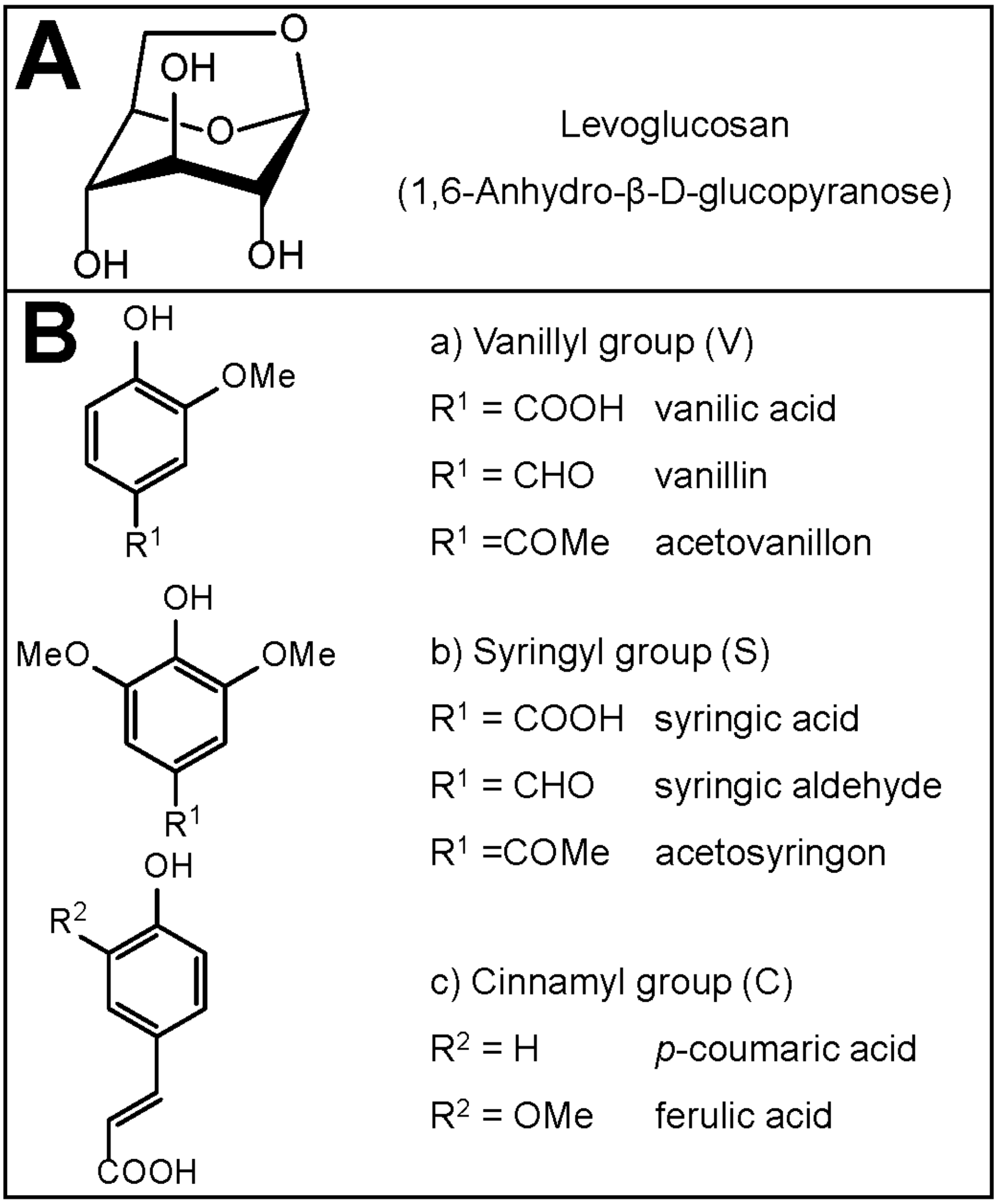

Supplementary Figure 1. Structural formulas of levoglucosan (A) and the eight investigated lignin oxidation products (LOPs) (B), sorted by structural group.

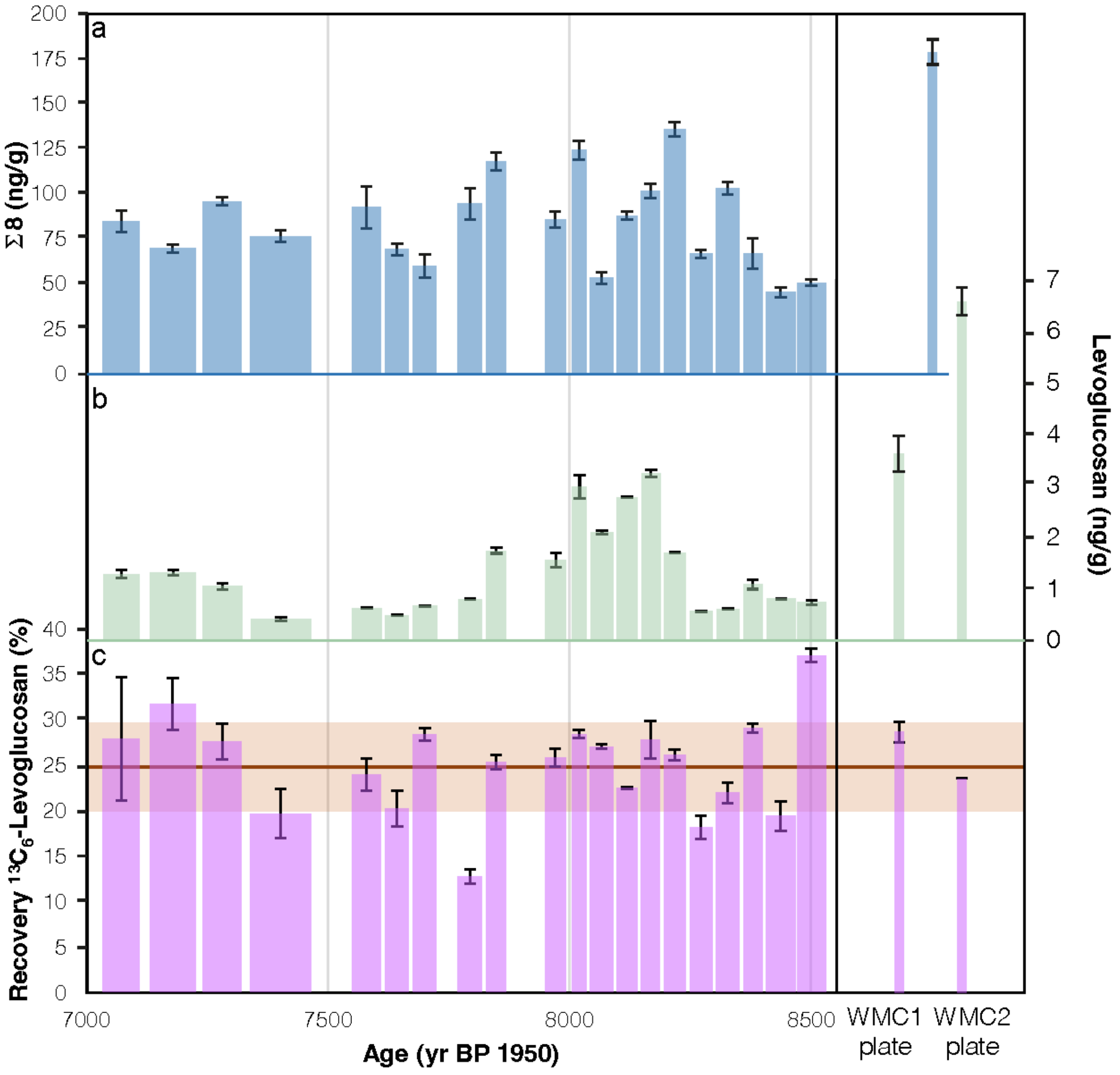

Supplementary Figure 2. Determined lignin oxidation products (LOPs), levoglucosan concentrations, and recovery rates of $^{13}C_6$ levoglucosan. a: Determined concentration of the sum of all 8 LOPs (Σ8; blue); b: Levoglucosan (light green); c: Determined recovery rates of $^{13}C_6$ levoglucosan (pink) of the 20 speleothem samples and the modern calcite samples. WMC1 plate was in place from March 2016 – June 2018 and WMC2 plate from December 2015 – June 2018. Width of the lines represent the time range for each sample. Error bars represent the standard deviation of samples measured in duplicate. Modern calcite samples were combined for the LOP measurements. The horizontal brown line indicates the average recovery for all speleothem samples and the shaded area represents the error, calculated as the standard deviation.

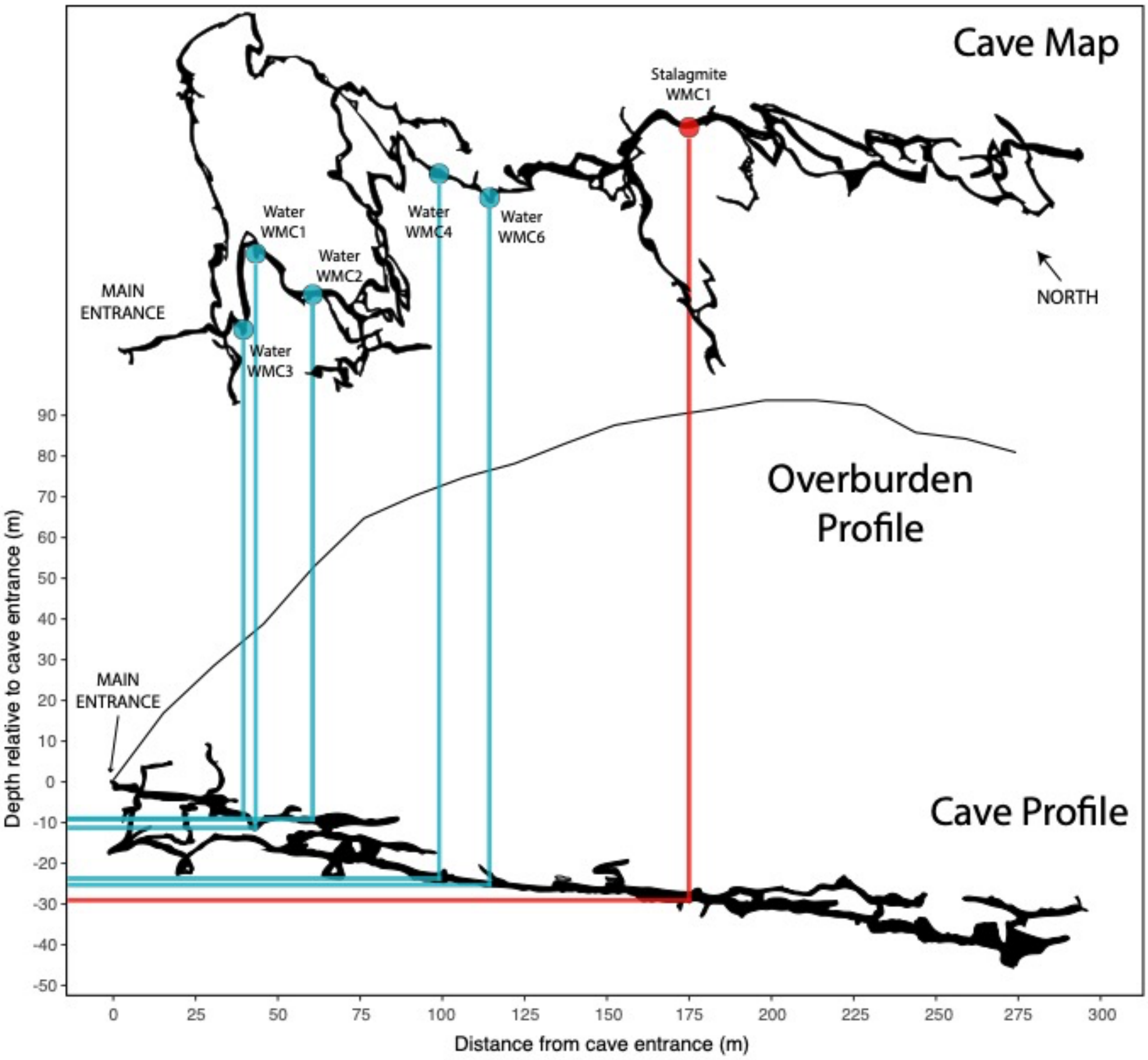

Supplementary Figure 3. Map of White Moon Cave (WMC) and approximated overburden profile. Water and modern calcite sampling sites are marked in blue; site of the investigated speleothem is marked in red. The overburden at the sample WMC1 is ca. 120 m.

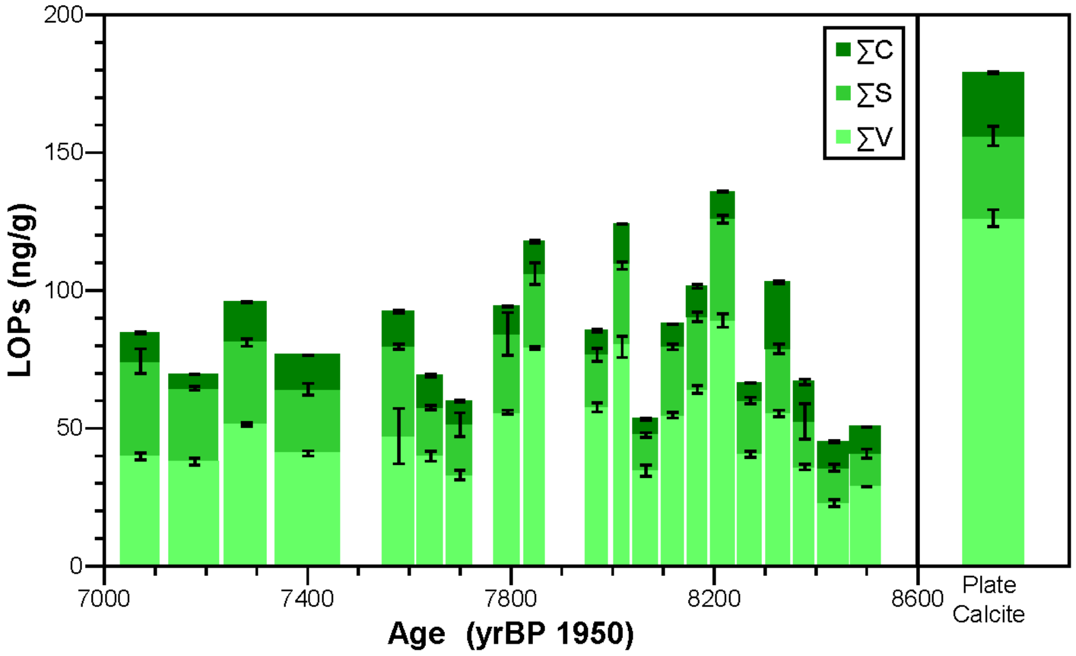

Supplementary Figure 4. Determined lignin oxidation products (LOPs) concentrations of the 20 speleothem samples and combined modern calcite samples shown by structural group. Light green: sum of the V-group LOPs; Green: sum of the S-group LOPs; Dark green: sum of the C-group LOPs. Error bars represent the standard deviation of samples measured in duplicate.

Supplementary *Table 1.* ***Ages of the speleothem and modern calcite samples, as well as determined levoglucosan concentration, $^{13}C_6$-levoglucosan recovery, and LOP concentrations***.

Sample	Depth from top (cm)	Age range (cal yr BP 1950)†	Levoglucosan ± SD (ng/g)	$^{13}C_6$-levoglucosan recovery ± SD (%)	∑V ± SD (ng/g)	∑S ± SD (ng/g)	∑C ± SD (ng/g)	∑8 ± SD (ng/g)
a*	5 to 5.5	<6900	4.74 ± 0.28	49.6 ± 1.8	95.78 ± 10.64	69.94 ± 11.12	27.26 ± 0.99	192.97 ± 22.75
b*	5.5 to 6.2		2.80 ± 0.13	45.6 ± 2.6	98.02 ± 2.81	54.80 ± 2.52	25.18 ± 1.46	178.00 ± 6.79
c*	6.25 to 6.9		2.21 ± 0.14	38.8 ± 0.1	77.41 ± 2.76	57.37 ± 5.99	19.64 ± 0.48	154.43 ± 9.24
d*	7 to 8		1.31 ± 0.01	36.9 ± 16.2	12.02 ± 0.29	8.40 ± 0.90	3.76 ± 0.11	24.19 ± 1.30
e	8 to 8.6	7071 ± 37	1.29 ± 0.08	27.9 ± 6.8	39.83 ± 1.23	34.58 ± 4.45	10.17 ± 0.43	84.58 ± 6.11
f	8.75 to 9.4	7178 ± 48	1.32 ± 0.06	31.7 ± 2.9	37.89 ± 1.28	26.65 ± 0.72	4.99 ± 0.06	69.53 ± 2.06
g	9.5 to 10.1	7280 ± 40	1.06 ± 0.06	27.6 ± 2.0	51.37 ± 0.58	29.70 ± 1.31	14.64 ± 0.25	95.71 ± 2.14
h	10.25 to 11.5	7401 ± 62	0.41 ± 0.03	19.7 ± 2.7	40.89 ± 0.93	23.28 ± 2.10	12.19 ± 0.07	76.37 ± 3.10
i	12.5 to 13.25	7579 ± 30	0.63 ± 0.01	24.0 ± 1.8	47.19 ± 10.04	32.42 ± 0.96	12.71 ± 0.44	92.32 ± 11.44
j	13.25 to 13.9	7642 ± 24	0.49 ± 0.01	20.2 ± 1.9	39.91 ± 1.82	17.59 ± 0.76	11.60 ± 0.57	69.10 ± 3.15
k	13.9 to 14.5	7700 ± 24	0.67 ± 0.01	28.4 ± 0.7	33.02 ± 1.76	18.33 ± 4.26	8.47 ± 0.33	59.81 ± 6.36
l	15 to 15.6	7793 ± 25	0.81 ± 0.003	12.7 ± 0.8	55.90 ± 0.64	28.39 ± 7.71	10.03 ± 0.25	94.31 ± 8.60
m	15.6 to 16.1	7847 ± 20	1.74 ± 0.06	25.3 ± 0.8	79.24 ± 0.48	26.84 ± 3.95	11.67 ± 0.60	117.74 ± 5.03
n	17 to 17.5	7970 ± 22	1.56 ± 0.14	25.8 ± 1.0	57.63 ± 1.64	19.07 ± 2.30	8.80 ± 0.57	85.50 ± 4.51
o	17.65 to 18	8019 ± 15	2.99 ± 0.22	28.4 ± 0.5	79.61 ± 3.88	29.37 ± 1.30	15.23 ± 0.09	124.21 ± 5.28
p	18.1 to 18.7	8066 ± 24	2.10 ± 0.04	27.1 ± 0.2	34.66 ± 2.01	12.94 ± 0.87	5.64 ± 0.32	53.24 ± 3.20
q	18.8 to 19.4	8118 ± 21	2.79 ± 0.004	22.5 ± 0.1	54.81 ± 1.01	24.70 ± 1.01	8.37 ± 0.10	87.88 ± 2.13
r	19.4 to 20	8167 ± 20	3.25 ± 0.07	27.8 ± 2.1	64.08 ± 1.44	26.38 ± 1.69	11.02 ± 0.67	101.48 ± 3.79
s	20 to 20.6	8217 ± 23	1.72 ± 0.01	26.2 ± 0.6	89.11 ± 2.43	36.78 ± 1.34	9.93 ± 0.21	135.82 ± 3.99
t	20.7. to 21.3	8271 ± 23	0.57 ± 0.01	18.2 ± 1.2	40.59 ± 1.09	19.56 ± 1.12	6.38 ± 0.10	66.53 ± 2.30
u	21.3 to 21.9	8327 ± 24	0.62 ± 0.003	22.0 ± 1.1	55.37 ± 1.24	23.44 ± 1.74	24.05 ± 0.51	102.87 ± 3.49
v	22 to 22.5	8379 ± 19	1.09 ± 0.09	29.1 ± 0.5	35.94 ± 0.98	16.63 ± 6.38	14.31 ± 1.04	66.88 ± 8.41
w	22.5 to 23.25	8436 ± 30	0.82 ± 0.01	19.4 ± 1.6	22.90 ± 1.32	12.76 ± 1.25	9.56 ± 0.29	45.22 ± 2.85
x	23.3 to 24	8500 ± 29	0.74 ± 0.04	37.1 ± 0.8	28.84 ± 0.13	12.03 ± 1.57	9.63 ± 0.16	50.50 ± 1.87
Mod A	/	/	3.62 ± 0.35	28.7 ± 1.2	126.63 ± 3.00	29.89 ± 3.56	23.05 ± 0.38	179.58 ± 6.94
Mod B	/	/	6.57 ± 0.27	23.6 ± 0.0				

*Results of measurements of these samples were not discussed in the manuscript as the dating on them is uncertain and no other proxy data is available for comparison.

† Ages provided are for the midpoint of the sample depth range ± the upper and lower bounds of the sample depth range.
